# Growth Pattern in Chinese Children With 5α-Reductase Type 2 Deficiency: A Retrospective Multicenter Study

**DOI:** 10.3389/fphar.2019.00173

**Published:** 2019-03-15

**Authors:** Xiu Zhao, Yanning Song, Shaoke Chen, Xiumin Wang, Feihong Luo, Yu Yang, Linqi Chen, Ruimin Chen, Hui Chen, Zhe Su, Di Wu, Chunxiu Gong

**Affiliations:** ^1^Center of Endocrinology, Genetics and Metabolism, National Center for Children’s Health, Beijing Children’s Hospital, Capital Medical University, Beijing, China; ^2^Department of Endocrinology, Shenzhen Children’s Hospital, Shenzhen, China; ^3^Genetic and Metabolic Central Laboratory, Maternal and Children Health Hospital of Guangxi Zhuang Autonomous Region, Nanning, China; ^4^Department of Endocrinology, Shanghai Children’s Medical Center, Shanghai Jiao Tong University, Shanghai, China; ^5^Department of Endocrinology, Children’s Hospital of Fudan University, Fudan University, Shanghai, China; ^6^Department of Endocrinology, Jiangxi Provincial Children’s Hospital, Nanchang, China; ^7^Department of Endocrinology, Children’s Hospital of Soochow University, Suzhou, China; ^8^Department of Endocrinology, Fuzhou Children’s Hospital, Fuzhou, China; ^9^Department of BME, Capital Medical University, Beijing, China

**Keywords:** dihydrotestosterone, testosterone, 5α-reductase type 2 deficiency, 46, XY DSD, height, children

## Abstract

**Background:**

5α-reductase type 2 deficiency (5αRD) is an autosomal recessive hereditary disease of the group of 46, XY disorders of sex development (DSD).

**Objective:**

To study the growth pattern in Chinese pediatric patients with 5αRD.

**Subjects:**

Data were obtained from 141 patients with 5αRD (age: 0–16 years old) who visited eight pediatric endocrine centers from January 2010 to December 2017.

**Methods:**

In this retrospective cohort study, height, weight, and other relevant data were collected from the multicenter hospital registration database. Baseline luteinizing hormone (LH), follicle stimulating hormone (FSH), testosterone (T), and dihydrotestosterone (DHT) after human chorionic gonadotropin (HCG) stimulation test were measured by enzyme enhanced chemiluminescence assay. Bone age (BA) was assessed using the Greulich-Pyle (G-P) atlas. Growth curve was constructed based on λ-median-coefficient of variation method (LMS).

**Results:**

The height standard deviation scores (HtSDS) and weight standard deviation scores (WtSDS) in 5αRD children were in the normal range as compared to normal boys. Significantly higher HtSDS was observed in patients with 5αRD who were <1 year old (*t* = 3.658, 2.103, *P* = 0.002, 0.048, respectively), and higher WtSDS in those <6 months old (*t* = 2.756, *P* = 0.012). Then HtSDS and WtSDS decreased gradually and fluctuated near the median of the same age until 13 years. WtSDS in 5αRD children from northern China were significantly higher than those from the south (*Z* = -2.670, *P* = 0.008). The variation tendency of HtSDS in Chinese 5αRDs was consistent with the trend of stimulating T. HtSDS and stimulating T in the external masculinization score (EMS) <7 group were slightly higher than those in EMS ≥ 7 group without significant difference. Additionally, the ratio of BA over chronological age (BA/CA) was significantly <1 in children with 5αRD.

**Conclusion:**

Children with 5αRD had a special growth pattern that was affected by high levels of T, while DHT played a very small role in it. Their growth accelerated at age <1 year, followed by slowing growth and fluctuating height near normal median boys’ height. The BA was delayed in 5αRD children. Androgen treatment, which may be considered anyway for male 5αRD patients with a micropenis, may also be beneficial for growth.

## HIGHLIGHTS

- The first growth curve in children with 5αRD.
- Our study provided evidence for applying androgen products at specific time periods for 5αRD patients with a micropenis.

## Introduction

Sex hormones are synthesized in the gonads and adrenal glands (Shen, 2016) and have bioactivity within bone and other target tissues (Vanderschueren et al., 2014). Their biological effects on bone are mediated by different cell types and mechanisms (Almeida et al., 2017) and controlled by gonadotropins via hypothalamic-pituitary feedback. Before puberty, longitudinal bone growth shows no significant sex differences (Nishiyama et al., 2012). During puberty, estrogen shows the biphasic regulation of longitudinal bone growth and epiphyseal closure. Early in puberty, estrogen at low concentrations can stimulate longitudinal growth via indirect effects on GH and insulin-like growth factor I (IGF-I), both of which stimulate growth plate chondrocytes (Veldhuis et al., 2005b, 2011; Almeida et al., 2017). However, in late puberty, higher levels of estrogen can exert inhibitory effects on the growth plate via estrogen receptors in the chondrocytes. Androgens mainly have direct effects on GH and show the influence on circulating IGF-I via peripheral and central aromatization (Veldhuis et al., 2005a, 2009). Whether androgen receptor in chondrocytes contributes to sex differences in longitudinal growth remains unclear. Thus, sex hormones have an essential role for male and female growth.

Disorders of sex development are defined as congenital conditions associated with atypical development of gonadal, chromosomal, or anatomical sex, such as androgen insensitivity syndrome (AIS) (Wang et al., 2017) and 5αRD. With the rapid development of next-generation sequencing and the popularity of precision medicine, DSD as one of rare diseases can be diagnosis earlier and more accurately (Jia and Shi, 2017; Ni and Shi, 2017). Furthermore, different DSDs may have different characteristics and different growth patterns partly owing to different changes in sex hormones. For example, gonadal dysplasia impacts physical development throughout the prenatal period until adulthood (Hughes et al., 2002; Richter-Unruh et al., 2004). In addition, height in children with CAIS who had their gonads removed in the pre-pubertal stage has shown to be lower than those who had it done after the puberty (Han et al., 2008), thus addressing the effect of sex hormones on pre-pubertal height. In our previous study, we found that children with 46, XY DSD are shorter than the normal population (Di et al., 2013). In addition, DSD children with T <100 ng/dL after HCG test are shorter than those with T ≥ 100 ng/dL (Wu et al., 2017). However, published literature on DSD growth is scarce.

5α-reductase type 2 deficiency (OMIM 264600) is an autosomal recessive hereditary disease with an incidence of 11.2–15.5% among patients with 46, XY DSD (Veiga-Junior et al., 2012; Ittiwut et al., 2017) and is caused by loss-of-function mutations of the SRD5A2 gene on chromosome 2. The mutations make the enzyme defective and impair T to DHT conversion (Sultan et al., 2001); therefore, individuals with 5αRD may develop malformation of external genitalia, including pseudovaginalis, ambiguous genitalia, hypospadias, micropenis, and cryptorchid or, in some cases, even a normal phenotype (Choi et al., 2008). Most of the patients with 5αRD are born as females, and more than 50% undergo gender self-reassignment during puberty (Wilson et al., 1993; Kolesinska et al., 2014; Bertelloni et al., 2016; Deeb et al., 2016). While many research studies have focused on gender reassignment (Deeb et al., 2016; Byers et al., 2017; Raveenthiran, 2017), our study investigates the growth and development of 5αRD patients. With the help of growth pattern, doctors can get more information for diagnosis and antidiastole of 5αRD. Perhaps, this will also help in accurate genetic analysis. Thus far, only two studies have reported on a 5αRD growth pattern in children. Ko et al. (2010) examined six Korean children with 5αRD and found a height percentile of P95, P90, and P90th in three cases that did not receive hormone treatment or gonadectomy. Eren et al. (2016) found the HtSDS being -0.31, +0.24, and +0.48 in three untreated Turkish children with 5αRD. In this systemic multicenter study, we aimed to determine the growth of 5αRD children.

## Materials and Methods

### Patients

All 187 patients with 5αRD (age 0–16 years) and admitted to eight pediatric endocrine centers from January 2010 to December 2017 were included in this retrospective cohort study. Their clinical manifestations included ambiguous genitalia at birth, hypospadias, micropenis, and cryptorchid. The ratio of T over dihydrotestosterone (T/DHT) after HCG stimulation test in all patients fluctuated between 10.67 and 86.56 (M: 32.72). All patients were diagnosed as 5αRD according to their manifestations and ratio of T/DHT >8.5 (Avendano et al., 2018). Then, the diagnosis was confirmed by pathogenic *SRD5A2* gene mutations. All patients had 46, XY karyotype and did not require hormonal treatment. The serum T level was >100 ng/dL after HCG stimulation test.

We excluded patients with malformation, abnormal functioning of the liver and kidney, or other with systemic diseases which may affect the physical development. Patients with 17β-hydroxysteroid dehydrogenase type 3, androgen insensitivity syndrome, and other types of 46, XY DSD were excluded by biochemical diagnosis and genetic confirmation. Informed consent for genetic testing was obtained from parents or caregivers of all study subjects.

In our study, we enrolled 141 cases and excluded 46 cases according to the inclusion and exclusion criteria. There were no significant differences in initial gender, age, geographical distribution, BWt, BL, BA, LH, FSH, T, and DHT levels after HCG stimulation test between inclusion and exclusion groups (*χ^2^* = 0.983, *P* = 0.321, *t* = 1.483, *P* = 0.140, *χ^2^* = 3.678, *P* = 0.055, *Z* = -0.864, *P* = 0.388, *Z* = -0.288, *P* = 0.773, *Z* = -1.700, *P* = 0.095, *Z* = -1.101, *P* = 0.271, *Z* = -0.206, *P* = 0.837, *Z* = -0.612, *P* = 0.540, *Z* = -0.164, *P* = 0.870, respectively). Also patients with EMS <7 showed no significantly different from those with EMR ≥ 7 between inclusion and exclusion groups (*χ^2^* = 2.841, *P* = 0.092).

### Data Collection

Data on anthropometry, CA, native place, gestational term, BL, BWt, family history, external genitalia and EMS (Ahmed et al., 2000), baseline LH and FSH, BA, T, and DHT levels after HCG stimulation test (Ishii et al., 2015) were recorded. Height was measured as orthostatic height when patients were aged >3 years. Patients aged <3 years were measured based on supine length. For each case, the height and weight were measured three times by experienced nurses, and the average value was considered.

### Outcome Measures

The following were main outcomes: HtSDS and WtSDS of different age groups were compared to the control subjects of the same age (Li et al., 2009).

We chose Chinese normal boys as the control group for the following reasons: All patients in the study had the 46, XY karyotype; about 60% of 5αRD children who were assigned the female gender in the period of infancy had marked masculinization and were reassigned as males at puberty (Lee et al., 2006).

### HCG Stimulation Test, BA, and Hormone Examination

Bone age, hormonal, and HCG stimulation test data were collected from the Beijing Children’s Hospital. Hormones were tested by enzyme enhanced chemiluminescence assay (Siemens Immulite 2000, Munich, Germany). BA was assessed according to the G-P atlas, by the same endocrinologist based on radiographic films of the left hand.

### Growth Curves Plotting

The internationally well accepted method (λ-median-coefficient of variation, LMS) for generating standard curves was adopted to calculate the M, S, and L (after converting the data into a normal distribution, using Box-Cox transformation) (Cole and Green, 1992), which described the growth index in each age band. L, M, and S of smooth curves and the required percentile were calculated using age as an independent variable. Growth curves (P3, P10, P25, P50, P75, P90, P97 percentile curves as well as -2SD, -1SD, 0SD, +1SD, +2SD standard deviation curves) for children in the age groups of 0–36 months and 3–13 years were constructed.

### Gene Analysis

All patients underwent genetic testing. Five to ten milliliters of peripheral blood was collected in disposable vacuum tubes for genetic testing. Genomic DNA was isolated using the QIAamp DNA Blood Mini Kit (Qiagen, Hilden, Germany) according to the manufacturer’s instructions. After construction according to the standard protocol, whole exon sequencing (100X) of the libraries was performed with SureSelect Human All Exon V6 array on the Illumina HiSeq X Ten Platform with PE150 strategy. To detect the potential variants in the cases, we performed bioinformatic processing and data analysis after receiving the primary sequencing data. Sequence variants were carefully identified with the help of GATK (McKenna et al., 2010) software following the best practice guidelines recommended by GATK (DePristo et al., 2011; Van der Auwera et al., 2013), including local realignment around INDELs, base quality score recalibration, followed by SNVs. INDELs were called simultaneously with the default setting of GATK Unified Genotyper on the realigned and recalibrated reads, followed by SNV and INDEL filtering to eliminate false-positive calls. The pathogenic SRD5A2 gene mutations were confirmed by homozygous or compound heterozygous mutations inherited from parents or *de novo*. Nucleotide sequences of SRD5A2 gene were compared with the published data. The pathogenicity of unreported SRD5A2 mutations was tested by *in silico* analysis using two software of “SIFT” (https://sift.bii.a-star.edu.sg/www/code.html) and “Polyphen” (http://genetics.bwh.harvard.edu/pph2/). The interpretation of gene pathogenicity is based on the ACMG (Richards et al., 2015).

### Data Analysis

The calculations for the growth curves were performed using LMS-chartmaker Pro software, and curves were drawn using the GraphPad Prism 6 software. SPSS 23.0 software was used for statistical analyses. Data pertaining to quantitative variables were expressed as mean ± SD or quartiles. Intergroup differences between two groups were assessed using the Student’s *t*-test for normally distributed data and Mann–Whitney *U*-test for non-normally distributed data. Multiple age group comparisons were assessed using the Kruskal–Wallis *H*-test and Bonferroni correction. The ratio of BA/CA was assessed using 95% CIs. Intergroup differences between inclusion and exclusion groups were assessed using the Chi-square test for qualitative data. *P* < 0.05 was considered statistically significant. Geographic difference analysis was based on north and south regions; Qinling Mountains and Huaihe River were selected as geographical boundaries between the south and north.

## Results

### General Data of Chinese Cases With 5αRD

The initial sex assignment was male in 143/187 (76.5%) and female in 44/187 (23.5%) cases. The clinical presentation was simple micropenis (*n* = 64, 34.2%); simple hypospadias (*n* = 43, 23.0%); ambiguous genitalia (*n* = 37, 19.8%); simple cryptorchid (*n* = 19, 10.2%); and others (micropenis and/or hypospadias and/or cryptorchid) (*n* = 24, 12.8%). Only two patients were siblings; the others were unrelated. The details of standard phenotype and gene mutations (Jia and Shi, 2017) were shown in the section of Supplementary Tables 1, 2).

**Figure 1:**
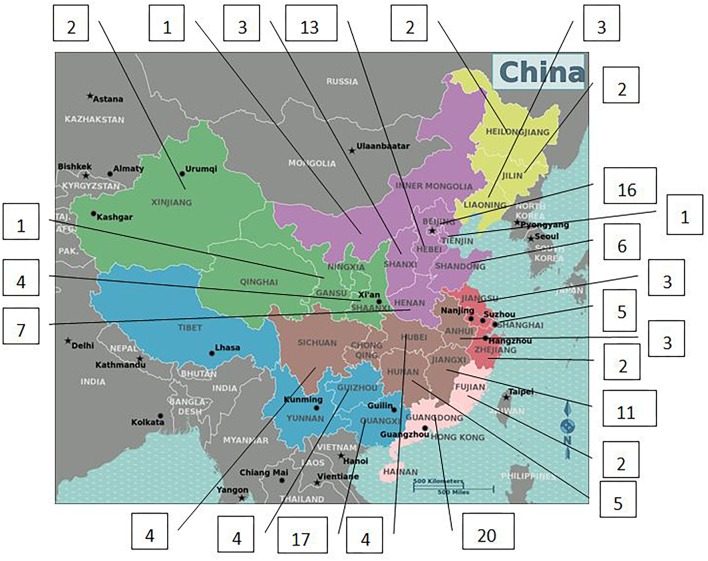
Geographical distribution of children with 5αRD in China.

**Figure 2:**
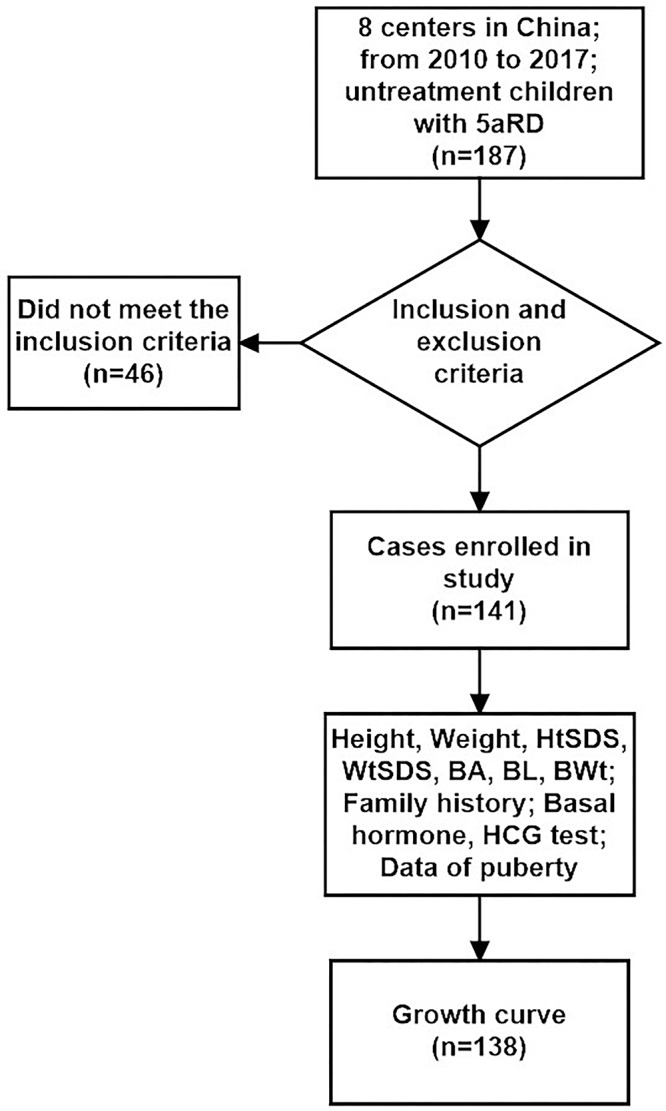
Flow chart of the study design.

Only 141 of 187 patients with 5αRD who had the data of height and weight and were aged between 0.08 and 16 years (M age: 1.75 years) were enrolled in our study. All 141 children with 5αRD came from 25 provinces and municipalities across China (Figure 1). Seven cases had presentations of puberty, with a testicular volume of ≥4 mL (Table 1). Four cases aged between 10 and 12 years (M age: 11.83 years) were in Tanner stage 2. The other 3 cases aged ≥13 years (M age: 15.16 years) were in Tanner stage 3–5. When plotting growth curve, we enrolled 138 cases aged <13 years because only 3 patients were aged between 13 and 16 years (Table 1, cases 5–7). Patients were divided into the following six groups: 0–5 months, 6–11 months, 1–2 years, 3–5 years, 6–9 years, and 10–12 years. The flow chart of the study is presented in Figure 2.

**Table 1:** Clinical parameters of children with 5aRD in the pubertal period.

No.	Age (year)	Ht (cm)	HtSDS	Wt (cm)	WtSDS	BMI (kg/m^2^)	BA (year)	Testes volume (ml)
1	11.17	143	-0.4	45.2	0.9	22.17	10.7	4
2	11.5	149	0	38	-0.3	17.16	12.6	4
3	12.17	148	-0.6	37	-0.7	16.94	–	5
4	12.83	152	-0.5	45.5	0	19.75	13	6
5	14.5	164	-0.6	49.5	-0.62	18.4	14	10
6	15	160	-1.5	44	-1.5	17.19	–	8
7	16	175	0.5	65	0.5	21.22	14	25

*Data of cases 1–4 were included for the growth curve. Data of cases 5–7 were excluded for the growth curve. Ht, height, Wt, weight; HtSDS, height standard deviation score; WtSDS, weight standard deviation score; BMI, body mass index; BA, bone age*.

### Physical Parameters of Chinese Children With 5αRD (Table 2)

**Table 2:** Physical assessments for Chinese children with 5αRD among different age groups.

Age groups	*n*	Age (year)	THtSDS	BWt (kg)	BL (cm)	HtSDS	WtSDS	BMI (kg/m^2^)
0–5 m	21	0.3 (0.3, 0.4)	0.1 ± 0.7	3.5 (3.2, 3.7)	50.0 (49.0, 50.0)	1.2 ± 1.4*^a^*	0.6 ± 1.1*^a^*	17.6 (16.9, 18.3)
6–11 m	23	0.7 (0.6, 0.9)	0.3 ± 0.6	3.3 (3.0, 3.8)	50.0 (49.0, 50.0)	0.5 ± 1.1*^a^*	0.3 ± 1.0	17.1 (16.1, 19.0)
1–2 y	48	1.8 (1.4, 2.2)	0.1 ± 0.8	3.5 (3.0, 3.8)	50.0 (49.0, 50.0)	-0.1 ± 1.2*^b^*	-0.1 ± 1.0	16.5 ± 1.4
3–5 y	24	3.8 (3.5, 4.8)	-0.1 (-0.6, 0.3)	3.2 (2.8, 3.5)	50.0 (49.0, 50.0)	-0.2 ± 1.3*^b^*	-0.2 ± 1.3	15.8 ± 1.8
6–9 y	11	7.1 (6.9, 9.0)	-0.4 (-0.9, 0.1)	3.4 (2.9, 3.6)	50.0 (48.0, 50.0)	0.2 ± 0.8	-0.1 ± 1.0	15.3 (13.4, 17.8)
10–12 y	11	11.3 (10.0, 12.2)	-0.1 ± 0.9	3.5 (3.1, 3.7)	50.0 (48.0, 50.5)	0.1 ± 0.5	-0.0 ± 0.7	17.9 ± 2.2
Total	138	1.8 (0.8, 3.8)	-0.08 (-0.50, 0.50)	3.4 (3.0, 3.7)	50.0 (49.0, 50.0)	0.2 ± 1.3	0.1 ± 1.0	16.9 (15.5, 17.8)
*P*	*^a^*					0.02, 0.048	0.012	

*P < 0.05, a vs. median of normal Chinese boys’ general reference values in the same age groups. b vs. 0–5 m group. THtSDS, target height standard deviation score; BWt, birth weight; BL, birth length; HtSDS, height standard deviation score; WtSDS, weight standard deviation score; BMI, body mass index; m, months; y, years*.

**Figure 3:**
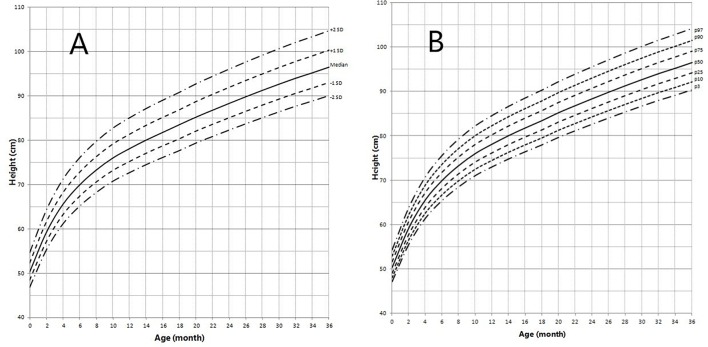
Height in children with 5αRD (0–36 months old). **(A)** Standard deviation curve; **(B)** Percentile curve.

**Figure 4:**
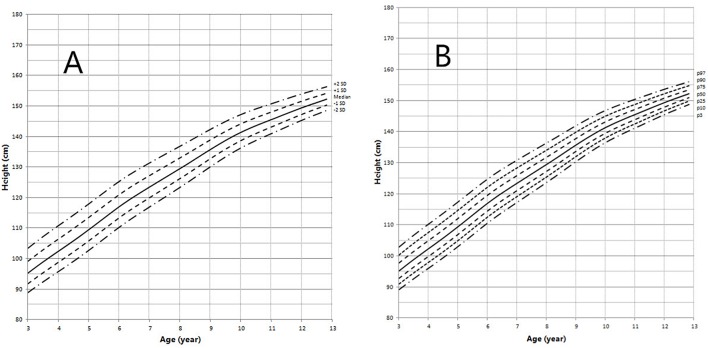
Height in children with 5αRD (3–13 years old). **(A)** Standard deviation curve; **(B)** Percentile curve.

**Figure 5:**
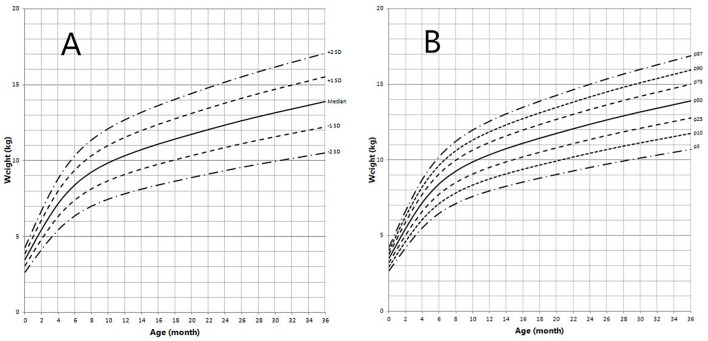
Weight in children with 5αRD (0–36 months old). **(A)** Standard deviation curve; **(B)** Percentile curve.

**Figure 6:**
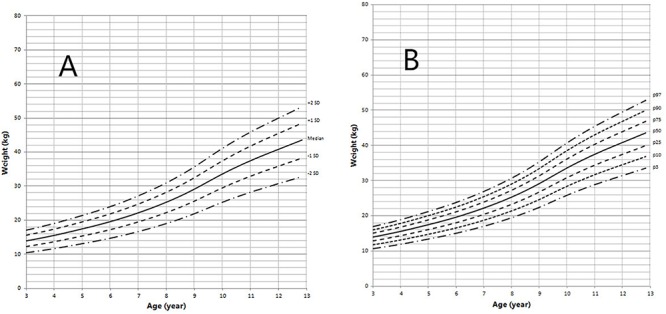
Weight in children with 5αRD (3–13 years old). **(A)** Standard deviation curve; **(B)** Percentile curve.

Height and weight curves are shown in Figure 3–6.

**Figure 7:**
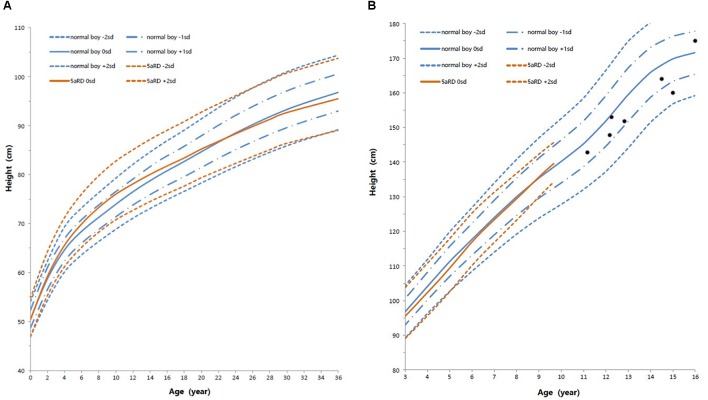
Standard deviation height curves between children with 5aRD and normal Chinese boys (0–16 years old). **(A)** Aged 0–36 months old; **(B)** Aged 3–16 years old. Red is 5αRD; Blue is the normal Chinese boy. Black spot is the patients of 5αRD older than 13 years. sd, standard deviation scores.

Height standard deviation scores and WtSDS of 138 cases with 5αRD were 0.21 ± 1.25 and 0.10 ± 1.02, respectively and in the normal range. The average BL of 5αRD was 50.00 (49.00, 50.00) cm and the average BWt of 5αRD was 3.40 (3.00, 3.70) kg, within the normal range, which was comparable to the normal reference standard for the Chinese population. Compared to the normal reference values for boys of the same age, HtSDS and WtSDS of 5αRD patients were higher when they were younger than 2 years old (the mean of HtSDS and WtSDS ranged from 0SD to +1SD) with significant difference aged <1 year in HtSDS (*t* = 3.658, 2.103, *P* = 0.002, 0.048; respectively) and aged <6 months in WtSDS (*t* = 2.756, *P* = 0.012). After that, their height and weight plateaued gradually and fluctuated around the median for normal Chinese boys until the age of 13 years (Figure 7). To sum up, these data showed that the height in 5αRD children increased faster than the normal population before 2 years of age, especially within the first 1 year of life, after which, the growth velocity gradually reduced and fluctuated near the median height of normal Chinese boys of the same age.

### Physical Assessments and Hormone Levels of 5αRD Among Different Age Groups

#### Physical Assessment for Chinese Children With 5αRD Among Different Age Groups (Table 2)

The highest and lowest HtSDS were found in the 0–5 months and 3–5 years groups. HtSDS in the 0–5 months group had significant differences with the 1–2 years and 3–5 years groups (*P* = 0.001, 0.004, respectively), while no significant differences were observed in the other groups.

The highest WtSDS was observed in the 0–5 months group; however, no significant differences were observed among the different age groups.

#### Hormones in Different Age Groups of 5αRD

The baseline levels of LH, FSH and the levels of T and DHT after HCG stimulation test are shown in Table 3. The lowest levels of LH, FSH, and T were found in the 3–5 years group. LH and FSH in the 0–5 months and 10–12 years groups were higher than the other age groups with significant differences to those in the 3–5 years group (*P* = 0.003, 0.048, 0.007, 0.009, respectively). The top three levels of T were observed in the 0–5 months, 6–11 months, and 10–12 years groups. Further, T in the 0–5 months and 6–11 months groups were significantly higher than in those aged 1–9 years (*P* = 0.001, 0.001, 0.003, 0.027, 0.002, 0.033, respectively). The lowest DHT level was seen in the 6–9 years group. The highest DHT was seen in the 0–5 months group and showed significant differences with those in the 6–9 years group (*P* = 0.008). The variation tendency of HtSDS in 5αRDs was consistent with the trend of T.

**Table 3:** Hormonal characterization for Chinese children with 5αRD among different age groups.

Agegroups	*n*	LH (mIU/ml)	FSH (mIU/ml)	T (ng/dl)	DHT (ng/ml)
0–5 m	14	1.88 (1.34, 3.12)	2.12 (1.32, 3.50)	489.68 ± 103.44	149.69 ± 35.56
6–11 m	14	0.34 (0.14, 3.0)	1.22 (0.91, 2.10)	448.18 ± 99.36	134.00 (101.14, 179.25)
1–2 y	36	0.27 (0.12, 0.95)	0.78 (0.43, 1.31)	331.01 ± 85.79^*a,c*^	127.30 (97.43, 164.08)
3–5 y	15	0.12 (0.10, 0.41)^*a,b*^	0.85 (0.65, 1.56)^*a,b*^	282.93 ± 70.91*^a,c^*	100.315 (70.83, 133.70)
6–9 y	9	0.29 (0.12, 1.44)	1.85 (0.50, 3.10)	287.63 ± 138.97^*a,c*^	69.90 (37.19, 101.05)^*a*^
10–12 y	7	1.20 (0.64, 3.79)	3.15 ± 1.57	360.03 ± 147.01	118.16 ± 51.75
Total	95	0.36 (0.13, 1.56)	1.27 (0.71, 2.30)	362.10 ± 121.60	123.00 (95.10,162.32)
*P*	*a*	0.003	0.048	0.001, 0.001, 0.003	0.008
	*b*	0.007	0.009		
	*c*			0.027, 0.002, 0.033	

*P < 0.05, a vs. 0–5 m group, b vs. 10–12 years group, and c vs. 6–11 m group. Testosterone (T) and dihydrotestosterone (DHT) were data after human chorionic gonadotropin stimulation test. LH, luteinizing hormone; FSH, follicle stimulating hormone; m, months; y, years*.

#### Ratio of BA/CA in Children With 5αRD (Table 4)

**Table 4:** BA/CA in children with 5αRD among different age groups.

Agegroups	BA/CA	95%CI	
		Lower	Upper
0–5 m	1.0 ± 0.32	0.7045	1.3197
6–11 m	0.77 ± 0.25	0.4256	1.0700
1–2 y	0.76 ± 0.23^c^	0.6611	0.8495
3–5 y	0.93 ± 0.21^c^	0.7962	0.9495
6–9 y	0.99 ± 0.04	0.8852	1.0882
10–12 y	0.93 ± 0.04	0.8279	1.0341
Total	0.88 ± 0.23^c^	0.8226	0.9359

*P < 0.05, c vs. 1. BA, bone age; CA, chronological age; m, months; y, years*.

The ratio of BA/CA (*N* = 68) was 0.88 ± 0.23 with 95% CI < 1. Additionally, 95% CI < 1 for BA/CA ratio were found in those aged 1–5 years, which meant that BA in 5αRDs aged between 1 and 5 years was delayed, then BA was almost near to CA in the other age groups.

### Physical Assessments and Hormone Levels of 5αRD According to EMS (Table 5)

**Table 5:** Physical assessments for Chinese children with 5αRD between two EMS groups.

EMS	n	BWt (kg)	BL (cm)	HtSDS	WtSDS	BMI (kg/m^2^)	T (ng/dL)	DHT (ng/mL)
<7	45	3.5 ± 0.5	50.0 (49.0, 50.0)	0.5 ± 1.5	0.2 ± 0.9	16.9 (15.4, 17.8)	363.3 (296.7, 415.4)	112.3 (73.1, 160.0)
≥7	50	3.3 (2.9, 3.7)*a*	49.5 (48.3, 50.0)	0.2 ± 1.0	0.0 ± 1.1	16.6 (15.2, 17.8)	350.7 ± 121.4	128.6 (99.4, 166.9)
*P*	*a*	0.028	0.083	0.350	0.388	0.758	0.371	0.166

*P < 0.05, a vs. 5αRDs with EMS < 7. Testosterone (T) and dihydrotestosterone (DHT) were data after human chorionic gonadotropin stimulation test. EMS, external masculinization score; BWt, birth weight; BL, birth length; HtSDS, height standard deviation score; WtSDS, weight standard deviation score; BMI, body mass index*.

Children with 5αRD were divided into two groups according to EMS (n = 95). One group was severely undervirilized with an EMS < 7, and the other was mildly undervirilized with an EMS ≥ 7. In the EMS < 7 group, HtSDS, WtSDS, BL, and T after HCG stimulation test were higher, and DHT after HCG stimulation test was lower than those in the EMS ≥ 7 group without significant differences. BWt in EMS < 7 group was significantly higher (Z = -2.191, P = 0.028).

### Geographical Difference in Children With 5αRD (Table 6)

**Table 6:** Clinical parameters of children with 5αRD in the north and south region of China.

Region	*n*	Age (year)	THtSDS	BWt (kg)	BL (cm)	HtSDS	WtSDS	BMI (kg/m^2^)	BA/CA	T ^(ng/dL)^	DHT (ng/mL)
South	77	2.00(0.86, 4.96)	-0.47 ± 1.74	3.30 ± 0.43	50(49, 50)	0.10 ± 1.24	-0.20(-0.80,0.50)	16.66 ± 2.27	0.89 ± 023	352.01 ± 151.20	103.00(63.61, 259.52)
North	61	1.42(0.68, 3.07)	0.25(-0.17, 0.75)*^a^*	3.44 ± 0.67*^a^*	50(49, 50)	0.20(-0.60, 1.00)	0.33 ± 1.08*^a^*	17.08(15.80, 17.78)	0.91(0.67, 1.03)	346.50(235.75, 491.50)	131.70(67.90, 240.18)
*P*	*a*	0.058	0.001	0.011	0.504	0.485	0.008	0.356	0.943	0.766	0.440

*P < 0.05, a South region vs. north region using Student’s t-test for normal distribution data and Mann–Whitney U-test for non-normal distribution data. T (testosterone) and DHT (dihydrotestosterone) were data after human chorionic gonadotropin stimulation test. THtSDS, target height standard deviation score; BWt, birth weight; BL, birth length; HtSDS, height standard deviation score; WtSDS, weight standard deviation score; BMI, body mass index; BA, bone age; CA, chronological age*.

Data indicated that THtSDS, BWt, and WtSDS in children with 5αRD from northern China were significantly higher than those from the southern region (*Z* = -4.556, -2.558, -2.670, *P* = 0.001, 0.011, 0.008, respectively), and no differences were found in age, BL, HtSDS, BMI, BA/CA, T, and DHT between the two groups.

## Discussion

5α-reductase type 2 deficiency is a 5α-reductase isoenzyme 2 formation defect caused by the SRD5A2 gene mutation. Its clinical profile ranges from 46, XY presented as complete female external genitalia to under masculinized male external genitalia (Sinnecker et al., 1996; Mendonca et al., 2016) such as enlarged clitoris, hypospadias, and micropenis (Ng et al., 1990; Gad et al., 1997; Silver and Russell, 1999; Nicoletti et al., 2005; Avendano et al., 2018). With the help of gene analysis, more and more 5αRD can be diagnosed in some cases with the mild phenotype (Wang et al., 2004; Nie et al., 2011). These give clinicians a chance to profile the growth of children with 5αRD. The growth pattern of 5αRD is becoming a concerned focus by clinicians.

### Height Features in Chinese Children With 5αRDs

In the present study, the plotted growth chart showed that the average HtSDS in 5αRD patients was within the range for normal reference values of Chinese boys. Children with 5αRD grow faster than normal boys before the age of 2 years, particularly when <1 year old. Between the ages of 2 and 13 years, their growth velocity decreases gradually and height fluctuates around the median height of normal Chinese boys. This trend was concordant with stimulating T fluctuation levels in 5αRD children in the growing age. 5αRD had decreased 5α-reductase type 2 enzymatic activity caused by SRD5A2 gene mutation. In 5αRDs, the lower the residual activity of 5α-reductase type 2 isoenzyme, the greater the severity of the manifestation and higher accumulation of T. Patients with severe undervirilized male external genitalia (EMS < 7) had slightly higher HtSDS, WtSDS, BL, and T after the HCG stimulation test and significantly higher BWt. All these hint at T having an impact on their growth. In 5αRD children, increased activity of the hypothalamic-pituitary-testicular axis during infancy would result in higher T levels because of minipuberty and diminished conversion to DHT. Thereafter, the slowed growth rate is a consequence of lower T during the quiescence of childhood before puberty. Previous studies have shown that growth in DSD children is associated with androgens (Hughes et al., 2002; Richter-Unruh et al., 2004; Han et al., 2008; Di et al., 2013; Wu et al., 2017). For example, Han et al. (2008) assessed the height in patients with CAIS and found that patients who underwent gonadectomy after adolescence or during adulthood were taller than those who underwent the same surgery in the pre-pubertal phase. The same conclusion was drawn when comparing with testicular dysfunction 46, XY DSD (Wu et al., 2017). This suggests that androgen affects growth in the pre-pubertal stage despite being undetectable in childhood. T and DHT are two types of androgen. T increases growth in association with a direct elevation of GH and an indirect elevation of IGF-I, respectively; the latter occurs due to estrogen by peripheral and central aromatization (Veldhuis et al., 2005a,b, 2009; Perry et al., 2008). DHT, on the other hand, could not up-regulate the function of hypothalamo-somatotrope-IGF-I axis directly (Veldhuis et al., 1997) and could not be aromatized to estrogen. There are two 5-alpha-reductase isoenzymes (Wilson, 1972). The type 1 isoenzyme is distributed in the bone, skeletal muscle, osteoblast-like cells, and a few other tissues (Wilson, 1972; Thigpen et al., 1993; Issa et al., 2002; Tria et al., 2004), whereas the type 2 isoenzyme is distributed in the prostate, seminal vesicle epididymis, medulla oblongata, and other tissues (Thigpen et al., 1993; Tria et al., 2004). The capability of T to induce biologic actions in bone depends on localized intraskeletal sex steroid hormone metabolism via type-1 5-alpha-reductase isoenzyme specially expressed in bone tissue (Yarrow et al., 2015). Furthermore, T surges in perinatal periods and consequent imprinting of the GH/IGF-I axis has important positive effects on the growth plate (Sims et al., 2006; Vanderschueren et al., 2014). In conclusion, growth of children with 5αRD was affected by high levels of T, while DHT plays a very minor role in their growth. Individuals with 5αRD have exhibited different residual activity of 5α-reductase. T treatment, which may be considered anyway for 5αRDs patients with micropenis, may also have an extra benefit on their growth.

### Bone Age Feature in 5αRDs

Bone age is the best index of growth potential ability. Sex hormones promote bone maturation, which, in different types of DSDs show special characteristics (Bertelloni et al., 2010). Short-term treatment with testosterone undecanoate (Andriol) did not change the BA in our previous study (Chen et al., 2012). The average ratio of BA/CA in this study of 5αRD children was lower than 1, particularly in the ages of 1–5 years. Perhaps, physiological androgen resistance during early childhood could explain the BA delay in presence of high T concentration among 5αRD children. This research addressed concerns related to treatment with androgen products on bone maturation and provided some supporting evidence for androgen therapy in childhood. Suitable T therapy may be beneficial for either micropenis or height growth among children with 5αRD.

### Puberty Feature in Chinese 5αRDs

Four of 11 cases aged 10–12 years (M age: 11.83 years) had the signs of puberty with Tanner stage 2. Another 3 cases aged >13 years (M age: 15.16 years) who were excluded from the growth curve were in Tanner stage 3–5. Perhaps these data showed that the onset time of puberty in children with 5αRD was somewhat more delayed than those of normal Chinese boys (M age: 10.55 years) (Pubertal Study Group of the Subspeciality Group of Endocrinologie, Hereditary and Matabolic Diseases, Society of Pediatrics, and Chinese Medical Association, 2010). Growth deceleration before puberty may partly explain the growth retardation in 5αRD during the age of 10–12 years.

### Study Limitation

We acknowledge that our study has some limitations. There were relatively few patients older than 10 years; thus, the growth pattern in children with 5αRD during the puberty period was limited. Seven children aged >10 years were in the pubertal stage with Tanner stage 2–5. These data partly reflected the onset time of puberty in children with 5αRD. We need more cases to validate the therapeutic opinions in the follow-up study. Owing to technical constraints in most Chinese labs hereto, serum LH, FSH, T, and DHT were measured by enzyme enhanced chemiluminescence assay and not as per the gold standard of mass spectrometry. Nonetheless, our hormone results had the value to assess the patients’ sex hormones, because there are several publications that have used the same testing methods, and enzyme enhanced chemiluminescence assay is still widely used to measure hormones in many countries currently. The levels of GH, IGF-I, and estrogens were not mentioned in the research due to lack of sufficient data in patients’ medical records.

## Conclusion

The growth curve of children with 5αRD revealed the special pattern affected by T, while DHT plays a very minor role in it. Our growth curve can provide the reference for clinical judgment of Chinese children with 5αRD. In addition, patients showed lagging BA. Furthermore, androgen treatment, which may be considered anyway for 5αRD patients with micropenis, may also be beneficial for their growth.

## Note

Number of cases with 5αRD in the present study


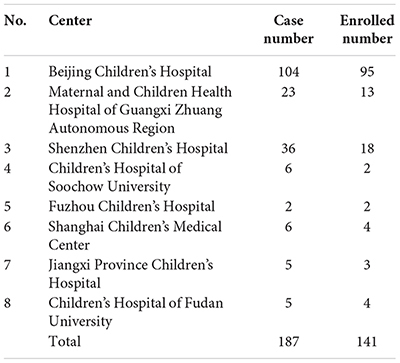


## Ethics Statement

This study was approved by the ethical committee of Beijing Children’s Hospital. This study was approved by the ethical committee of Beijing Children’s Hospital. All subjects gave written informed consent in accordance with the Declaration of Helsinki.

## Author Contributions

XZ contributed to data collection, data interpretation, and writing of the report. CG contributed to study design and reviewed the paper. YS, SC, XW, FL, YY, LC, RC, DW, and ZS contributed to data collection. HC contributed to statistical analysis.

## Conflict of Interest Statement

The authors declare that the research was conducted in the absence of any commercial or financial relationships that could be construed as a potential conflict of interest.

## Abbreviations

ACMG: American College of Medical Genetics and Genomics
BA: bone age
BL: birth length
BMI: body mass index
BWt: birth weight
CA: chronological age
CAIS: complete androgen insensitivity syndrome
CIs: confidence intervals
DHT: dihydrotestosterone
DSD: disorders of sex development
EMS: external masculinization score
5αRD: 5α-reductase type 2 deficiency
FSH: follicle stimulating hormone
GATK: genome analysis toolkit
GH: growth hormone
G-P: Greulich-Pyle
HCG: human chorionic gonadotropin
HtSDS: height standard deviation score
IGF-I: insulin-like growth factor-I
L: coefficient of skewness
LH: luteinizing hormone
LMS: λ-median-coefficient of variation method
M: median
S: coefficient of variation
SD: standard deviation
SNVs: single nucleotide variations
T: testosterone
THtSDS: target height standard deviation score
WtSDS: weight standard deviation score.
