# Supplementary material for: Growth Pattern in Chinese Children With 5α-Reductase Type 2 Deficiency: A Retrospective Multicenter Study
Source: Front Pharmacol. 2019 Mar 15;10:173. doi: 10.3389/fphar.2019.00173 (PMC6429988; doi:10.3389/fphar.2019.00173)
Supplement: Supplementary file 1 [file Table_1.docx]

Table 1. Phenotype of 187 Chinese children with 5α-reductase type 2 deﬁciency (5αRD)

| No. | Initial gender | Age | Family history | Clinical manifestation | | | | PHt | MHt | BWt | BL | Ht | Wt |
| --- | --- | --- | --- | --- | --- | --- | --- | --- | --- | --- | --- | --- | --- |
|  |  | (year) |  | Micropenis | Hypospadia | Cryptorchid | Ambiguous  genitalia | (cm) | (cm) | (kg) | (cm) | (cm) | (kg) |
| 1 | female | 1.75 | - | + | + | + |  | 176.00 | 155.00 | 2.70 | 48.00 | 87.90 | 11.30 |
| 2 | male | 2.00 | - | + | + |  |  | 164.00 | 163.00 | 2.90 | 50.00 | 89.80 | 13.30 |
| 3 | male | 3.00 | - | + |  | + |  | 172.00 | 158.00 | 3.50 | 49.00 | 93.70 | 12.20 |
| 4 | male | 7.00 | - | + | + |  |  | 178.00 | 167.00 | 3.30 | 50.00 | 120.30 | 25.30 |
| 5 | male | 1.25 | - | + |  | + |  | 160.00 | 156.00 | 3.80 | 48.00 | 76.40 | 9.70 |
| 6 | male | 9.00 | - | + | + |  |  | 167.00 | 158.00 | 3.40 | 48.00 | 137.00 | 23.50 |
| 7 | male | 7.00 | - | + | + |  |  | 174.00 | 153.00 | 2.50 | 48.00 | 127.20 | 21.60 |
| 8 | male | 10.00 | - | + | + |  |  | 168.00 | 161.00 | 3.90 | 51.00 | 139.30 | 32.60 |
| 9 | male | 3.75 | - | + |  |  |  | 174.00 | 152.00 | 2.50 | 47.00 | 105.50 | 15.30 |
| 10 | male | 2.17 | - | + | + |  |  | 169.00 | 157.00 | 2.60 | 48.00 | 87.80 | 12.80 |
| 11 | male | 1.75 | - | + |  |  |  | 181.00 | 165.00 | 3.10 | 49.00 | 89.10 | 12.40 |
| 12 | male | 1.67 | - | + |  |  |  | 170.00 | 151.00 | 2.70 | 50.00 | 82.50 | 9.10 |
| 13 | male | 3.50 | - | + |  | + |  | 169.00 | 156.00 | 3.40 | 49.00 | 102.30 | 15.10 |
| 14 | male | 3.92 | - | + |  |  |  | 173.00 | 153.00 | 3.60 | 49.00 | 103.70 | 14.90 |
| 15 | male | 0.33 | - | + |  |  |  | 165.00 | 150.00 | 3.60 | 50.00 | 59.80 | 6.40 |
| 16 | male | 11.25 | - | + | + |  |  | 178.00 | 162.00 | 2.50 | 47.00 | 147.00 | 38.00 |
| 17 | male | 2.17 | - | + |  |  |  | 167.00 | 153.00 | 2.70 | 47.00 | 91.50 | 14.10 |
| 18 | male | 0.42 | - | + | + |  |  | 169.00 | 156.00 | 3.10 | 48.00 | 67.00 | 8.70 |
| 19 | male | 8.33 | - | + |  |  |  | 170.00 | 153.00 | 3.60 | 48.00 | 128.90 | 25.90 |
| 20 | male | 0.92 | - | + |  |  |  | 171.00 | 158.00 | 3.00 | 49.00 | 78.00 | 9.60 |
| 21 | male | 6.42 | - | + |  |  |  | 173.00 | 154.00 | 2.80 | 50.00 | 127.30 | 20.60 |
| 22 | male | 6.92 | - | + |  |  |  | 167.00 | 152.00 | 3.60 | 50.00 | 125.60 | 22.40 |
| 23 | male | 1.00 | - | + |  |  |  | 164.00 | 160.00 | 2.80 | 50.00 | 78.80 | 9.55 |
| 24 | male | 3.00 | - | + | + |  |  | 173.00 | 154.00 | 2.90 | 50.00 | 81.80 | 10.50 |
| 25 | male | 0.67 | - | + |  |  |  | 168.00 | 160.00 | 3.50 | 49.00 | 72.30 | 7.60 |
| 26 | male | 1.75 | - | + |  |  |  | 169.00 | 155.00 | 3.30 | 50.00 | 80.70 | 11.30 |
| 27 | male | 1.09 | - | + |  |  |  | 172.00 | 157.00 | 2.80 | 48.00 | 77.80 | 11.10 |
| 28 | male | 11.17 | - | + | + |  |  | 163.00 | 151.00 | 3.10 | 50.00 | 142.80 | 45.20 |
| 29 | male | 0.42 | - | + |  |  |  | 172.00 | 153.00 |  |  | 65.00 | 7.45 |
| 30 | male | 0.42 | - | + | + |  |  | 166.00 | 157.00 | 2.60 | 48.00 | 57.00 | 6.55 |
| 31 | male | 9.75 | - | + | + |  |  | 174.00 | 162.00 | 3.70 | 50.00 | 139.00 | 36.20 |
| 32 | male | 5.92 | - | + |  |  |  | 163.00 | 158.00 | 2.90 | 50.00 | 121.60 | 28.40 |
| 33 | male | 3.92 | - | + |  |  |  | 175.00 | 164.00 | 3.20 | 50.00 | 97.70 | 14.20 |
| 34 | male | 1.33 | - | + | + |  |  | 175.00 | 170.00 | 3.80 | 50.00 | 87.00 | 13.00 |
| 35 | male | 5.00 | - | + |  |  |  | 173.00 | 160.00 | 3.30 | 49.00 | 108.50 | 19.00 |
| 36 | male | 3.25 | - | + |  |  |  | 178.00 | 161.00 | 3.60 | 50.00 | 100.00 | 16.00 |
| 37 | male | 1.08 | - | + |  |  |  | 170.00 | 163.00 | 4.20 | 50.00 | 78.50 | 10.00 |
| 38 | male | 1.08 | - | + |  |  |  | 170.00 | 163.00 | 2.80 | 49.00 | 75.00 | 9.50 |
| 39 | male | 1.75 | - | + |  |  |  | 169.00 | 162.00 | 3.10 | 49.00 | 73.00 | 10.00 |
| 40 | male | 0.08 | - | + |  |  |  | 181.00 | 159.00 | 3.80 | 53.00 | 55.00 | 3.90 |
| 41 | male | 12.25 | - | + |  |  |  | 170.00 | 155.00 | 3.70 | 50.00 | 153.00 | 40.00 |
| 42 | male | 0.25 | - | + |  |  |  | 170.00 | 165.00 | 4.00 | 50.00 | 62.00 | 6.00 |
| 43 | male | 0.60 | - | + |  |  |  | 178.00 | 167.00 | 3.80 | 50.00 | 95.00 | 9.20 |
| 44 | male | 1.00 | - | + |  |  |  | 178.00 | 160.00 | 3.10 | 50.00 | 75.00 | 9.00 |
| 45 | male | 0.80 | - | + |  |  |  | 168.00 | 164.00 | 3.65 | 50.00 | 75.00 | 9.40 |
| 46 | male | 0.75 | - |  |  | + |  | 180.00 | 170.00 | 4.00 | 51.00 | 92.00 | 16.20 |
| 47 | male | 4.42 | - | + | + |  |  | 180.00 | 156.00 | 2.80 | 47.00 | 103.50 | 16.00 |
| 48 | male | 0.23 | - |  |  | + |  | 169.00 | 160.00 | 3.30 | 49.00 | 58.00 | 6.30 |
| 49 | male | 4.42 | - |  |  | + |  | 169.00 | 160.00 | 3.50 | 50.00 | 105.00 | 19.00 |
| 50 | male | 0.42 | - |  |  | + |  | 175.00 | 168.00 | 3.60 | 50.00 | 72.50 | 9.00 |
| 51 | male | 0.42 | - | + | + |  |  | 170.00 | 158.00 | 3.50 | 50.00 | 70.00 | 8.40 |
| 52 | female | 3.67 | - |  |  |  | + | 178.00 | 170.00 | 2.80 | 49.00 | 97.00 | 14.00 |
| 53 | female | 1.33 | - |  |  |  | + | 170.00 | 162.00 | 3.70 | 48.00 | 80.00 | 10.00 |
| 54 | female | 1.50 | - |  |  |  | + | 165.00 | 154.00 | 3.70 | 50.00 | 80.00 | 10.50 |
| 55 | male | 2.17 | - | + | + |  |  | 160.00 | 163.00 | 3.30 | 49.00 | 92.00 | 13.50 |
| 56 | female | 2.00 | - |  |  |  | + | 169.00 | 159.00 | 3.70 | 50.00 | 81.50 | 12.00 |
| 57 | female | 0.23 | - |  |  |  | + | 183.00 | 165.00 | 3.60 | 51.00 | 64.30 | 6.63 |
| 58 | female | 0.79 | - |  |  |  | + | 172.00 | 155.00 | 3.20 | 49.00 | 69.50 | 8.20 |
| 59 | male | 2.40 | - | + |  |  |  | 170.00 | 159.00 | 2.86 | 49.00 | 86.00 | 15.00 |
| 60 | female | 3.50 | - |  |  |  | + | 172.00 | 160.00 | 3.05 | 50.00 | 102.00 | 18.00 |
| 61 | female | 2.00 | - |  |  |  | + | 176.00 | 163.00 | 3.35 | 49.00 | 90.00 | 14.00 |
| 62 | female | 2.60 | - |  |  |  | + | 178.00 | 165.00 | 3.50 | 49.00 | 82.50 | 11.50 |
| 63 | male | 0.33 | - | + |  |  |  | 175.00 | 160.00 | 3.45 | 50.00 | 72.00 | 8.87 |
| 64 | male | 1.42 | - | + |  |  |  | 168.00 | 154.00 | 3.70 | 49.00 | 77.00 | 9.00 |
| 65 | female | 1.00 | - |  |  |  | + | 170.00 | 165.00 | 2.30 | 49.00 | 74.50 | 9.50 |
| 66 | female | 0.17 | - |  |  |  | + | 178.00 | 160.00 | 3.20 | 48.00 | 61.00 | 6.00 |
| 67 | female | 0.58 | - |  |  |  | + | 173.00 | 165.00 | 2.90 | 50.00 | 70.00 | 10.00 |
| 68 | female | 0.50 | - |  |  |  | + | 163.00 | 160.00 | 3.25 | 50.00 | 75.00 | 10.00 |
| 69 | male | 0.42 | - | + |  |  |  | 169.00 | 160.00 | 3.25 | 50.00 | 70.00 | 9.00 |
| 70 | female | 5.58 | - |  |  |  | + | 159.00 | 161.00 | 2.70 | 50.00 | 110.00 | 17.00 |
| 71 | female | 2.50 | - |  |  |  | + | 168.00 | 165.00 | 3.80 | 52.00 | 92.00 | 15.00 |
| 72 | male | 1.50 | - | + |  |  |  | 170.00 | 165.00 | 3.25 | 50.00 | 79.00 | 11.00 |
| 73 | female | 1.25 | - |  |  |  | + | 165.00 | 150.00 | 3.00 | 50.00 | 82.00 | 12.00 |
| 74 | female | 3.70 | - |  |  |  | + | 172.00 | 153.00 | 3.60 | 52.00 | 105.00 | 22.00 |
| 75 | male | 1.90 | - | + |  |  |  | 163.00 | 155.00 | 3.60 | 50.00 | 81.00 | 11.50 |
| 76 | female | 3.25 | - |  |  |  | + | 178.00 | 154.00 | 4.50 | 52.00 | 103.90 | 14.00 |
| 77 | female | 0.92 | - |  |  |  | + | 170.00 | 167.00 | 3.80 | 49.00 | 78.00 | 11.00 |
| 78 | female | 2.13 | - |  |  |  | + | 166.00 | 156.00 | 4.00 | 52.00 | 90.00 | 13.50 |
| 79 | male | 1.95 | - | + |  |  |  | 176.00 | 162.00 | 3.10 | 52.00 | 85.60 | 12.50 |
| 80 | female | 0.50 | - |  |  |  | + | 177.00 | 160.00 | 3.20 | 49.00 | 67.00 | 7.90 |
| 81 | female | 9.00 | - |  |  |  | + | 167.00 | 152.00 | 3.50 | 50.00 | 135.00 | 25.00 |
| 82 | female | 0.33 | - |  |  |  | + | 171.00 | 152.00 | 2.50 | 47.00 | 65.00 | 8.00 |
| 83 | female | 0.25 | - |  |  |  | + | 169.00 | 164.00 | 3.70 | 52.00 | 58.50 | 6.00 |
| 84 | male | 1.58 | - |  |  |  | + | 172.00 | 167.00 | 3.90 | 50.00 | 83.50 | 12.00 |
| 85 | male | 1.42 | - | + |  |  |  | 165.00 | 158.00 | 3.50 | 50.00 | 84.00 | 10.50 |
| 86 | male | 1.08 | - | + |  |  |  | 173.00 | 165.00 | 3.80 | 52.00 | 80.00 | 11.50 |
| 87 | female | 2.00 | - |  |  |  | + | 173.00 | 158.00 | 3.40 | 49.00 | 87.50 | 13.00 |
| 88 | male | 4.50 | - | + |  |  |  | 175.00 | 160.00 | 3.80 | 49.00 | 105.00 | 16.80 |
| 89 | male | 1.42 | - | + |  |  |  | 165.00 | 158.00 | 3.50 | 50.00 | 84.00 | 10.50 |
| 90 | male | 3.47 | silbing | + |  |  |  | 174.00 | 158.00 | 2.50 | 48.00 | 98.00 | 16.00 |
| 91 | female | 2.25 | sibling |  |  |  | + | 178.00 | 168.00 | 3.80 | 50.00 | 97.00 | 15.00 |
| 92 | male | 10.60 | - | + |  |  |  | 178.00 | 168.00 | 3.70 | 50.00 | 144.00 | 30.00 |
| 93 | male | 1.46 | - | + |  |  |  | 170.00 | 170.00 | 4.20 | 50.00 | 81.50 | 12.00 |
| 94 | male | 0.33 | - | + |  |  |  | 178.00 | 158.00 | 4.10 | 50.00 | 62.00 | 6.46 |
| 95 | female | 1.46 | - |  |  |  | + | 174.00 | 164.00 | 3.50 | 50.00 | 82.00 | 12.00 |
| 96 | male | 2.75 | - | + |  |  |  | 175.00 | 155.00 | 3.75 | 52.00 | 97.00 | 14.50 |
| 97 | male | 12.17 | - | + |  |  |  |  |  |  |  | 147.80 | 37.00 |
| 98 | male | 0.58 | - | + |  |  |  |  |  | 2.70 | 48.00 | 65.50 | 7.40 |
| 99 | male | 0.42 | - | + |  |  |  |  |  | 3.50 | 54.00 | 66.00 | 7.80 |
| 100 | male | 0.67 | - | + |  |  |  |  |  | 3.15 | 51.00 | 71.00 | 8.20 |
| 101 | female | 1.33 | - |  |  |  | + |  |  | 3.05 | 50.00 | 76.00 | 10.50 |
| 102 | male | 6.00 | - | + |  |  |  |  |  | 2.70 | 50.00 | 105.00 | 18.50 |
| 103 | male | 5.50 | - | + |  |  |  |  |  | 3.25 | 50.00 | 93.00 | 13.00 |
| 104 | male | 4.25 | - | + |  |  |  |  |  | 3.00 |  | 94.00 | 15.00 |
| 105 | male | 0.67 | - |  | + | + |  |  |  | 3.00 |  | 69.50 | 9.20 |
| 106 | female | 3.00 | - |  | + | + |  |  |  |  |  | 95.00 | 13.50 |
| 107 | male | 1.33 | - |  | + |  |  |  |  | 2.95 | 51.00 | 78.00 | 9.80 |
| 108 | male | 10.00 | - |  | + |  |  |  |  |  |  | 117.60 | 22.00 |
| 109 | male | 10.00 | - |  | + |  |  |  |  |  |  | 147.00 | 42.00 |
| 110 | female | 3.50 | - |  |  |  | + | 165.00 | 155.00 |  |  | 92.00 | 12.80 |
| 111 | male | 12.83 | - |  | + |  |  | 170.00 | 160.00 |  |  | 151.80 | 45.50 |
| 112 | male | 1.17 | - |  | + |  |  | 173.00 | 160.00 |  |  | 82.80 | 11.92 |
| 113 | male | 2.16 | - |  | + |  |  | 160.00 | 159.00 | 3.00 | 49.00 | 84.00 | 9.59 |
| 114 | male | 6.75 | - |  | + |  |  | 160.00 | 159.00 | 3.75 | 50.00 | 115.00 | 20.20 |
| 115 | male | 2.47 | - |  | + |  |  | 164.00 | 163.00 | 3.50 | 50.00 | 90.50 | 12.70 |
| 116 | male | 3.07 | - |  | + |  |  | 167.00 | 158.00 | 3.10 | 50.00 | 90.00 | 15.00 |
| 117 | female | 4.83 | - |  |  |  | + | 173.00 | 160.00 | 3.50 | 50.00 | 108.00 | 18.50 |
| 118 | male | 0.76 | - |  | + |  |  | 175.00 | 158.00 | 3.30 | 48.00 | 75.00 | 11.50 |
| 119 | female | 11.50 | - |  |  |  | + | 170.00 | 160.00 | 3.50 |  | 148.80 | 38.00 |
| 120 | female | 1.58 | - |  |  |  | + |  |  | 3.40 | 50.00 | 78.80 | 10.00 |
| 121 | male | 3.07 | - |  | + |  |  | 165.00 | 152.70 | 2.75 |  | 95.00 | 12.00 |
| 122 | female | 2.25 | - |  |  |  | + | 162.00 | 162.00 | 3.40 |  | 88.00 | 12.00 |
| 123 | male | 0.92 | - |  | + |  |  | 172.00 | 160.00 | 4.15 | 53.00 | 79.50 | 12.00 |
| 124 | male | 0.25 | - |  | + |  |  | 170.00 | 158.00 | 3.60 | 50.00 | 63.30 | 7.98 |
| 125 | male | 0.50 | - |  | + |  |  | 168.00 | 158.00 | 3.75 | 50.00 | 69.00 | 8.50 |
| 126 | male | 0.58 | - |  | + |  |  | 168.00 | 163.00 | 3.30 | 50.00 | 69.00 | 8.10 |
| 127 | male | 0.50 | - |  | + |  |  |  |  |  |  | 68.00 | 8.20 |
| 128 | male | 1.58 | - |  | + |  |  | 163.00 | 153.00 | 3.50 | 50.00 | 75.00 | 10.50 |
| 129 | male | 1.00 | - |  | + |  |  | 170.00 | 159.00 | 3.80 | 49.00 | 77.00 | 9.70 |
| 130 | male | 0.42 | - |  | + |  |  | 179.00 | 167.00 | 4.20 | 51.00 | 70.00 | 8.50 |
| 131 | female | 7.08 | - |  |  |  | + |  |  | 2.90 |  | 130.20 | 45.00 |
| 132 | male | 0.50 | - |  |  | + |  | 173.00 | 158.00 | 2.50 | 48.00 | 78.50 | 13.00 |
| 133 | male | 11.83 | - |  |  | + |  | 158.00 | 157.00 | 3.25 |  | 133.70 |  |
| 134 | male | 0.50 | - |  |  | + |  | 172.00 | 147.00 | 3.15 |  | 68.50 | 8.30 |
| 135 | male | 9.42 | - |  |  | + |  |  |  | 3.00 | 48.00 | 134.10 | 32.00 |
| 136 | male | 0.50 | - |  | + |  |  | 180.00 | 160.00 | 3.95 | 51.00 | 68.00 | 8.80 |
| 137 | female | 0.83 | - | + | + |  |  |  |  | 4.00 |  | 73.00 | 9.00 |
| 138 | male | 3.17 | - |  | + |  |  | 174.00 |  | 3.50 | 50.00 | 104.00 | 17.50 |
| 139 | male | 14.00 | - |  |  | + |  |  |  |  |  |  |  |
| 140 | male | 3.17 | - |  | + |  |  |  |  |  |  |  |  |
| 141 | female | 1.17 | - |  |  | + |  |  |  |  |  |  |  |
| 142 | male | 5.00 | - |  |  | + |  |  |  |  |  |  |  |
| 143 | male | 10.00 | - |  |  | + |  |  |  |  |  |  |  |
| 144 | female | 3.00 | - |  |  | + |  |  |  |  |  |  |  |
| 145 | male | 13.08 | - |  | + |  |  |  |  |  |  |  |  |
| 146 | male | 7.00 | - |  | + |  |  |  |  |  |  |  |  |
| 147 | female | 0.75 | - |  |  |  | + |  |  |  |  |  |  |
| 148 | female | 14.5 | - | + |  | + |  |  |  |  |  | 164.00 | 49.5 |
| 149 | male | 3.00 | - |  | + |  |  |  |  |  |  |  |  |
| 150 | male | 3.00 | - |  | + |  |  |  |  |  |  |  |  |
| 151 | male | 2.00 | - |  | + |  |  |  |  |  |  |  |  |
| 152 | male | 15.00 | - |  |  | + |  |  |  |  |  | 160.00 | 44.00 |
| 153 | male | 16.00 | - |  | + |  |  |  |  |  |  | 175.00 | 65.00 |
| 154 | male | 0.67 | - |  |  | + |  |  |  |  |  |  |  |
| 155 | female | 8.00 | - |  |  | + |  |  |  |  |  |  |  |
| 156 | male | 0.08 | - |  | + |  |  |  |  |  |  |  |  |
| 157 | female | 4.50 | - |  |  | + |  |  |  |  |  |  |  |
| 158 | male | 0.42 | - |  | + |  |  |  |  |  |  |  |  |
| 159 | male | 1.58 | - |  | + |  |  |  |  |  |  |  |  |
| 160 | male | 9.00 | - |  | + |  |  |  |  |  |  |  |  |
| 161 | male | 3.58 | - |  | + |  |  |  |  |  |  |  |  |
| 162 | male | 2.75 | - |  | + |  |  |  |  | 3.25 |  |  |  |
| 163 | male | 0.00 | - |  | + |  |  |  |  | 3.45 |  |  |  |
| 164 | female | 14.00 | - |  |  |  | + |  |  |  |  |  |  |
| 165 | male | 0.50 | - |  | + |  |  |  |  | 2.55 |  |  |  |
| 166 | male | 2 | - |  | + |  |  |  |  |  |  |  |  |
| 167 | male | 7 | - |  | + |  |  |  |  |  |  |  |  |
| 168 | male | 0.67 | - |  | + |  |  |  |  |  |  |  |  |
| 169 | male | 8 | - |  | + |  |  |  |  |  |  |  |  |
| 170 | male | 0.08 | - |  | + |  |  |  |  |  |  |  |  |
| 171 | male | 4.5 | - |  |  | + |  |  |  |  |  |  |  |
| 172 | male | 0.42 | - |  | + |  |  |  |  |  |  |  |  |
| 173 | male | 1.58 | - |  | + |  |  |  |  |  |  |  |  |
| 174 | male | 3 | - |  |  | + |  |  |  |  |  |  |  |
| 175 | male |  | - | + |  |  |  |  |  |  |  |  |  |
| 176 | male | 0.17 | - | + |  |  |  |  |  | 3.35 | 50.00 |  |  |
| 177 | male | 0.50 | - | + |  |  |  |  |  | 3.15 |  |  |  |
| 178 | male | 2.17 | - | + |  |  |  |  |  |  |  |  |  |
| 179 | male | 7.08 | - | + |  |  |  |  |  | 2.90 |  |  |  |
| 180 | male | 0.5 | - | + |  |  |  |  |  | 2.50 | 48.00 |  |  |
| 181 | male | 11.83 | - | + |  |  |  |  |  | 3.25 |  |  |  |
| 182 | male | 0.50 | - | + |  |  |  |  |  | 3.15 |  |  |  |
| 183 | male | 9.42 | - | + |  |  |  |  |  | 3.00 | 48.00 |  |  |
| 184 | male | 0.50 | - | + |  |  |  |  |  | 3.95 | 51.00 |  |  |
| 185 | male | 0.83 | - | + |  |  |  |  |  | 4.00 |  |  |  |
| 186 | male | 3.17 | - | + |  |  |  |  |  | 3.50 | 50.00 |  |  |
| 187 | male | 2.25 | - | + |  |  |  |  |  | 3.40 |  |  |  |

*PHt=paternal height, MHt=maternal height, BWt=birth weight, BL=birth length, Ht=height, Wt=weight, +=positive, -=negative.*
