# Supplementary material for: Growth Pattern in Chinese Children With 5α-Reductase Type 2 Deficiency: A Retrospective Multicenter Study
Source: Front Pharmacol. 2019 Mar 15;10:173. doi: 10.3389/fphar.2019.00173 (PMC6429988; doi:10.3389/fphar.2019.00173)
Supplement: Supplementary file 2 [file Table_2.docx]

Table 2. Gene mutations of 187 Chinese children with 5α-reductase type 2 deﬁciency (5αRD)

| No. | Allele | Mutation-1 | Mutation-1 | Mutation-2 | Mutation-2 | Mutation-3 | Mutation-3 |
| --- | --- | --- | --- | --- | --- | --- | --- |
| 1 | 1 | c.350C>A | p.A117D | c.542C>T | p.P181L |  |  |
| 2 | 1 | c.680G>A | p.R227Q | c.59T>C | p.L20P |  |  |
| 3 | 1 | c.680G>A | p.R227Q | c.737G>A | p.R246Q |  |  |
| 4 | 1 | c.680G>A | p.R227Q | c.211C>T | p.Q71* |  |  |
| 5 | 1 | c.737G>A | p.G203S | c.680G>A | p.R227Q | c.265C>G | p.L89V |
| 6 | 1 | c.16C>T | p.Q6* | c.680G>A | p.R227Q |  |  |
| 7 | 1 | c.607G>A | p.G203S | c.586G>A | p.G196S |  |  |
| 8 | 1 | c.265C>G | p.L89L | c.680G>A | p.R227Q |  |  |
| 9 | 1 | c.211C>T | p.Q71* | c.680G>A | p.R227Q |  |  |
| 10 | 0 | c.607G>A | p.G203S |  |  |  |  |
| 11 | 1 | c.680G>A | p.R227Q | c.737G>A | p.G203S |  |  |
| 12 | 0 | c.680G>A | p.R227Q |  |  |  |  |
| 13 | 1 | c.680G>A | p.R227Q | c.694C>G | p.H232D | c.265C>G | p.L89V |
| 14 | 1 | c.680G>A | p.R227Q | c.548-9T>G |  |  |  |
| 15 | 1 | c.578A>G | p.N193S | c.680G>A | p.R227Q |  |  |
| 16 | 0 | c.680G>A | p.R227Q |  |  |  |  |
| 17 | 0 | c.680G>A | p.R227Q |  |  |  |  |
| 18 | 1 | c.607G>A | p.G203S | c.679C>T | p.R227* | c.265C>G | p.L89V |
| 19 | 1 | c.680G>A | p.R227Q | c.680G>A | p.R227Q |  |  |
| 20 | 1 | c.679C>T | p.R227* | c.577A>G | p.I193V | c.265C>G | p.L89V |
| 21 | 1 | c.680G>A | p.R227Q | c.607G>A | p.G203S |  |  |
| 22 | 1 | c.680G>A | p.R227Q | c.737G>A | p.R246Q | c.265C>G | p.L89V |
| 23 | 1 | c.679C>T | p.R227* | c.607G>A | p.G203S |  |  |
| 24 | 1 | c.680G>A | p.R227Q | c.680G>A | p.R227Q |  |  |
| 25 | 1 | c.680G>A* | p.R227Q | c.737G>A | p.G203S |  |  |
| 26 | 1 | c.680G>A | p.R227Q | c.211C>T | p.Q71* |  |  |
| 27 | 1 | c.680G>A | p.R227Q | c.548-9T>G |  |  |  |
| 28 | 1 | c.680G>A | p.R227Q | c.736G>A | p.R246W |  |  |
| 29 | 1 | c.680G>A | p.R227Q | c.16C>T | p.Q6* |  |  |
| 30 | 1 | c.680G> | p.R227Q | c.694C>G | p.H232D |  |  |
| 31 | 1 | c.607G>A | p.G203S | c.680G>A | p.R227Q |  |  |
| 32 | 1 | c.680G>A | p.R227Q | c.586G>A | p.G196S |  |  |
| 33 | 1 | c.680G>A | p.R227Q | c.737G>A | p.R246Q |  |  |
| 34 | 1 | c.736C>T | p.R246W | c.679G>A | p.E227K |  |  |
| 35 | 1 | c.679G>A | p.E227K | c.577A>G | p.I193V |  |  |
| 36 | 1 | c.680G>A | p.E227K | c.16C>T | p.Q6* |  |  |
| 37 | 1 | c.680G>A | p.R227Q | c.607G>A | p.G203S |  |  |
| 38 | 1 | c.736G>A | p.R246W | c.16C>T | p.Q6* |  |  |
| 39 | 1 | c.679G>A | p.E227K | c.16C>T | p.Q6* |  |  |
| 40 | 1 | c.16C>T | p.Q6* | c.737G>A | p.R246Q |  |  |
| 41 | 1 | c.679G>A | p.E227K | c.100G>C | p.E34R |  |  |
| 42 | 1 | c.736G>A | p.R246Q | c.16C>T | p.Q6* |  |  |
| 43 | 0 | c.607G>A | p.G203S |  |  |  |  |
| 44 | 0 | c.736G>A | p.R246W |  |  |  |  |
| 45 | 1 | c.607G>A | p.G203S | c.59T>C | p.L20P |  |  |
| 46 | 1 | c.679G>A | p.E227K | c.104G>C | p.S35T |  |  |
| 47 | 1 | c.281-2A>G |  | c.89_90insC | p.P30fs |  |  |
| 48 | 1 | c.632T>A | p.L211P | c.289+9612_289+9613insAC |  |  |  |
| 49 | 1 | c.736G>A | p.R246W | c.679G>A | p.E227K |  |  |
| 50 | 1 | c.736G>A | p.R246W | c.679G>A | p.E227K |  |  |
| 51 | 1 | c.606G>A | p.G203S | c.16C>T | p.Q6* |  |  |
| 52 | 1 | c.100G>C | p.G34R | c.680G>A | p.R227Q |  |  |
| 53 | 1 | c.606G>A | p.G203S | c.89_90insC | p.P30fs |  |  |
| 54 | 1 | c.737G>A | p.R246Q | c.16C>T | p.Q6* |  |  |
| 55 | 1 | c.350C>A | p.A117D | c.542C>T | p.P181L |  |  |
| 56 | 1 | c.680G>A | p.R227Q | c.737G>A | p.R246Q |  |  |
| 57 | 1 | c.679G>A | p.E227K | c.577A>G | p.I193V |  |  |
| 58 | 0 | c.680G>A | p.R227Q |  |  |  |  |
| 59 | 1 | c.680G>A | p.R227Q | c.*91_c.*92in* |  |  |  |
| 60 | 1 | c.680G>A | p.R227Q | c.737G>A | p.R246Q |  |  |
| 61 | 0 | c.577A>G | p.I193V |  |  |  |  |
| 62 | 1 | c.577A>G | p.N193S | c.679G>A | p.E227K |  |  |
| 63 | 0 | c.16C>T | p.Q6* |  |  |  |  |
| 64 | 1 | c.679G>A | p.E227K | c.16C>T | p.Q6* |  |  |
| 65 | 1 | c.679G>A | p.E227K | c.170G>C | p.S57T |  |  |
| 66 | 1 | c.679G>A | p.E227K | c.16C>T | p.Q6* |  |  |
| 67 | 1 | c.577A>G | p.I193V | c.16C>T | p.Q6* |  |  |
| 68 | 0 | c.680G>A | p.R227Q |  |  |  |  |
| 69 | 1 | c.16C>T | p.Q6* | c.736G>A | p.G246R |  |  |
| 70 | 1 | c.680G>A | p.R227Q | c.16C>T | p.Q6* |  |  |
| 71 | 1 | c.105C>A | p.K35N | c.680G>A | p.R227Q |  |  |
| 72 | 0 | c.307G>A | p.S103X |  |  |  |  |
| 73 | 1 | c.679G>A | p.E227K | c.*91_c.*92E5insATATATATATATATATATAT |  |  |  |
| 74 | 1 | c.16C>T | p.Q6* | c.679G>A | p.E227K |  |  |
| 75 | 1 | c.16C>T | p.Q6* | c.607G>A | p.G203S |  |  |
| 76 | 0 | c.737G>A | p.R246Q |  |  |  |  |
| 77 | 0 | c.296C>T | p.S99L |  |  |  |  |
| 78 | 1 | c.680G>A | p.R227Q | c.683C>T | p.A228V |  |  |
| 79 | 1 | c.*91_c.*92insATATATATATATATATATAT |  | c.679G>A | p.E227K |  |  |
| 80 | 1 | c.680G>A | p.R227Q | c.433C>G | p.R145W |  |  |
| 81 | 1 | c.547-9T>G |  | c.737G>A | p.R246Q |  |  |
| 82 | 1 | c.736C>T | p.R246W | c.737G>A | p.R246Q |  |  |
| 83 | 1 | c.680G>A | p.R227Q | c.16C>T | p.Q6* |  |  |
| 84 | 1 | c.16C>T | p.Q6* | c.737G>A | p.R226Q |  |  |
| 85 | 1 | c.607G>A | p.G203S | c.680G>A | p.R227Q |  |  |
| 86 | 1 | c.371T>A | p.V124D | c.681G>A | p.R327Q |  |  |
| 87 | 1 | c.607G>A | p.G203S | c.680G>A | p.R227Q |  |  |
| 88 | 1 | c.16C>T | p.Q6* | c.419G>A | p.W140* |  |  |
| 89 | 1 | c.268C>T | p.H90Y | c.680G>A | p.R227Q |  |  |
| 90 | 1 | c.371T>A | p.V124D | c.680G>A | p.R227Q |  |  |
| 91 | 1 | c.737G>A | p.P246Q | c.680G>A | p.R227Q |  |  |
| 92 | 0 | c.736G>A | p.R246Q |  |  |  |  |
| 93 | 1 | c.736G>A | p.R246Q | c.680G>A | p.R227Q |  |  |
| 94 | 1 | c.16C>T | p.Q6* | c.441C>T | p.S147= |  |  |
| 95 | 1 | c.*91_c.*92ins* |  | c.736C>T | p.R246W |  |  |
| 96 | 1 | c.737G>A | p.R246Q | c.680G>A | p.R227Q |  |  |
| 97 | 1 | c.89_90insC | p.P30fs | c.307G>A | p.S103X |  |  |
| 98 | 1 | c.89_90insC | p.P30fs | c.371T>A | p.V124D |  |  |
| 99 | 1 | C.682C>T | p.L228F | c.16C>T | P.Q6* |  |  |
| 100 | 0 | c.16C>T | p.Q6* |  |  |  |  |
| 101 | 0 | c.16C>T | p.Q6* |  |  |  |  |
| 102 | 1 | c.680G>A | p.R227Q | c.607G>A | G203S |  |  |
| 103 | 1 | c.680G>A | p.R227Q | c.307G>A | p.S103X |  |  |
| 104 | 1 | c.679G>A | p.E227K | c.679G>A | p.E227K |  |  |
| 105 | 1 | c.680G>A | p.R227Q | c.16C>T | p.Q6* |  |  |
| 106 | 1 | c.607G>A | p.G203S | c.737G>A | p.R246Q |  |  |
| 107 | 0 | c.680G>A | p.R227Q |  |  |  |  |
| 108 | 1 | c.680G>A | p.R227Q | c.296C>T | p.S99L |  |  |
| 109 | 1 | c.680G>A | p.R227Q | c.737G>A | p.R246Q |  |  |
| 110 | 1 | c.737G>A | p.R246Q | c.16G>T | p.Q6* |  |  |
| 111 | 1 | c.281+9T>C |  | c.296C>T | p.S99L |  |  |
| 112 | 1 | c.680G>A | p.R227Q | c.268C>T | p.H90Y | c.265C>G | p.L89V |
| 113 | 1 | c.680G>A | p.R227Q | c.607G>A | p.G203S |  |  |
| 114 | 0 | c.680G>A | p.R227Q |  |  |  |  |
| 115 | 0 | c.680G>A | p.R227Q |  |  |  |  |
| 116 | 1 | c.680G>A | p.R227Q | c.652delT | p.Phe219Sfs |  |  |
| 117 | 0 | c.680G>A | p.R227Q | c.679C>T | p.R227* |  |  |
| 118 | 0 | c.680G>A | p.R227Q |  |  |  |  |
| 119 | 1 | c.680G>A | p.R227Q |  |  |  |  |
| 120 | 1 | c.607G>A | p.G203S | c.50T>C | p.L17S |  |  |
| 121 | 0 | c.680G>A | p.R227Q |  |  |  |  |
| 122 | 1 | c.680G>A | p.R227Q | c.542C>T | p.P181L | c.265C>G | p.L89V |
| 123 | 1 | c.680G>A | p.R227Q | c.607G>A | p.G203S |  |  |
| 124 | 0 | c.680G>A | p.R227Q |  |  |  |  |
| 125 | 1 | c.680G>A | p.R227Q | c.737G>A | p.R246Q |  |  |
| 126 | 1 | c.680G>A | p.R227Q | c.725A>G | p.W246C |  |  |
| 127 | 0 | c.680G>A | p.R227Q |  |  |  |  |
| 128 | 1 | c.680G>A | p.R227Q | c.1331G>C | p.G444A |  |  |
| 129 | 0 | c.680G>A | p.R227Q |  |  |  |  |
| 130 | 1 | c.680G>A | p.R227Q | c.50T>C | p.L17S |  |  |
| 131 | 0 | c.680G>A | p.R227Q |  |  |  |  |
| 132 | 1 | c.680G>A | p.R227Q | c.656delT | p.F219fs |  |  |
| 133 | 1 | c.607G>A | p.G203S | c.374T>G | p.L125R |  |  |
| 134 | 1 | c.16C>T | p.Q6* | c.680G>A | p.R227Q |  |  |
| 135 | 1 | c.680G>A | p.R227Q | c.607G>A | p.G203S |  |  |
| 136 | 1 | c.680G>A | p.R227Q | c.607G>A | p.G203S |  |  |
| 137 | 0 | c.607G>A | p.G203S |  |  |  |  |
| 138 | 1 | c.680G>A | p.R227Q | c.663_664delTT | p.C221Ffs*10 |  |  |
| 139 | 1 | c.680G>A | p.R227Q | c.737G>A | p.R246Q |  |  |
| 140 | 1 | c.680G>A | p.R227Q | c.737G>A | p.R246Q |  |  |
| 141 | 1 | c.282-2A>G | p.? | c.725A>G | p.W246C |  |  |
| 142 | 1 | c.50T>C | p.L17S | c.680G>A | p.R227Q |  |  |
| 143 | 0 | c.16C>T | p.Q6* |  |  |  |  |
| 144 | 1 | c.16C>T | p.Q6* | c.737G>A | p.R246Q |  |  |
| 145 | 1 | c.680G>A | p.R227Q | c.1331G>C | p.G444A |  |  |
| 146 | 1 | c.656delT | p.F219fs | c.571G>A | p.G191R |  |  |
| 147 | 0 | c.623C>T | p.T208I |  |  |  |  |
| 148 | 1 | c.607G>A | p.G203S | c.680G>A | p.R227Q |  |  |
| 149 | 1 | c.737G>A | p.R246Q | c.680G>A | p.R227Q |  |  |
| 150 | 1 | c.737G>A | p.R246q | c.1331G>C | p.G444A |  |  |
| 151 | 1 | c.419_421delinsATTC | p.W140Wfs*8 | c.680G>A | p.R227Q |  |  |
| 152 | 1 | c.680G>A | p.R227Q | c.350C>A | P.A117D |  |  |
| 153 | 1 | c.607G>A | p.G203S | c.737G>A | p.R246Q |  |  |
| 154 | 1 | c.755dupT | p.I253Hfs*8 | c.607G>A | p.G203S |  |  |
| 155 | 1 | c.680G>A | p.R227Q | c.211C>T | p.Q71* |  |  |
| 156 | 1 | c.269A>C | p.H90P | c.680G>A | p.R227Q |  |  |
| 157 | 1 | c.16C>T | p.Q6* | c.100G>C | p.G34R |  |  |
| 158 | 1 | c.350C>A | p.A117D | c.680G>A | p.R227Q |  |  |
| 159 | 1 | c.16C>T | p.Q6* | c.680G>A | p.R227Q |  |  |
| 160 | 1 | c.737G>A | p.R246Q | c.268C>T | p.H90Y | c.265C>G | p.L89V |
| 161 | 1 | c.680G>A | p.R227Q | c.737G>A | p.R246Q |  |  |
| 162 | 1 | c.680G>A | p.R227Q | c.656delT | p.F219fs | c.265C>G | p.L89V |
| 163 | 1 | c.737G>A | p.R246Q | c.16G>T | p.Q6* |  |  |
| 164 | 0 | c.680G>A | p.R227Q |  |  |  |  |
| 165 | 1 | c.680G>A | p.R227Q | c.607G>A | p.G203S |  |  |
| 166 | 0 | c.680G>A | p.R227Q |  |  |  |  |
| 167 | 0 | c.680G>A | p.R227Q |  |  |  |  |
| 168 | 1 | c.680G>A | p.R227Q | c.652delT | p.F219fs |  |  |
| 169 | 0 | c.680G>A | p.R227Q |  |  |  |  |
| 170 | 1 | c.680G>A | p.R227Q | c.350C>A | p.A117D | c.265C>G | p.L89V |
| 171 | 0 | c.680G>A | p.R227Q |  | P.A22fs |  |  |
| 172 | 1 | c.16C>T | p.Q6* | c.281+15T>C | / |  |  |
| 173 | 1 | c.265C>G | p.L89V | c.211C>T | p.Q71* |  |  |
| 174 | 1 | c.265C>G | p.L89V | c.680G>A | p.R227Q |  |  |
| 175 | 1 | c.548-9T>G | / | c.737G>A | p.R246Q |  |  |
| 176 | 1 | c.265C>G | p.L89V | c.571G>A | p.G191R |  |  |
| 177 | 1 | c.16C>G | p.Q6* | c.680G>A | p.R227Q | c.265C>G | p.L89V |
| 178 | 1 | c.296C>T | p.S99L | c.656delT | p.F219fs | c.265C>G | p.L89V |
| 179 | 1 | c.737G>A | p.R246q | c.281G>A | p.R94K |  |  |
| 180 | 1 | c.680G>A | p.R227Q | c.281G>A | p.R94K |  |  |
| 181 | 1 | c.607G>A | p.G203S | c.16C>G | p.Q6* |  |  |
| 182 | 1 | c.680G>A | p.R227Q | c.350C>A | p.A117D |  |  |
| 183 | 0 | c.737G>A | p.R246Q |  |  |  |  |
| 184 | 1 | c.350C>A | p.A117D | c.6565delT | p.E219Sfs*？ |  |  |
| 185 | 0 | c.680G>A | p.R227Q |  |  |  |  |
| 186 | 0 | c.419G>A | p.W140* |  |  |  |  |
| 187 | 0 | c.268C>T | p.H90Y |  |  |  |  |

*0= homozygous mutations, 1= heterozygous mutations.*
